# Mast Cells and Angiogenesis in Human Plasma Cell Malignancies

**DOI:** 10.3390/ijms20030481

**Published:** 2019-01-23

**Authors:** Domenico Ribatti, Roberto Tamma, Angelo Vacca

**Affiliations:** 1 Department of Basic Medical Sciences, Neurosciences and Sensory Organs, University of Bari Medical School, 70124 Bari, Italy; roberto.tamma@uniba.it; 2 Department of Biomedical Sciences, and Human Oncology, Section of Internal Medicine and Clinical Oncology, University of Bari Medical School, 70124 Bari, Italy

**Keywords:** angiogenesis, anti-angiogenesis, mast cells, multiple myeloma, plasmocytoma

## Abstract

Bone marrow angiogenesis plays an important role in the pathogenesis and progression of hematological malignancies. It is well known that tumor microenvironment promotes tumor angiogenesis, proliferation, invasion, and metastasis, and also mediates mechanisms of therapeutic resistance. An increased number of mast cells has been demonstrated in angiogenesis associated with hematological tumors. In this review we focused on the role of mast cells in angiogenesis in human plasma cell malignancies. In this context, mast cells might act as a new target for the adjuvant treatment of these tumors through the selective inhibition of angiogenesis, tissue remodeling and tumor-promoting molecules, permitting the secretion of cytotoxic cytokines and preventing mast cell-mediated immune suppression.

## 1. Mast Cells in Human Plasma Cell Malignancies

Multiple myeloma (MM), solitary plasmocytoma of bone, and solitary extramedullary plasmocytoma belong to a spectrum of disorders referred to as plasma cell dyscrasias [1]. Solitary plasmocytoma of the bone represent an early stage of MM and patients with an apparent solitary lesion may have an occult MM [2], and solitary plasmocytoma of the skull base tend to progress to MM [3].

Mast cells represent a dominant infiltrate in human plasma cell malignancies, and the degree of mast cell infiltration parallels the severity of disease. Mast cells are a source of different cytokines, including interleukin-1, -2 and -6 (IL-1, IL-2, IL-6) and stem cells factor (SCF), all of which can induce plasma cell proliferation. IL-6 is the major plasma cell growth factor acting through both a paracrine and autocrine growth stimulation mechanism [4]. Addition of SCF to MM cell lines enhances the proliferation of myeloma cells and the response to IL-6 [5].

## 2. Mast Cells and Tumor Growth

Mast cells attracted in the tumor microenvironment by SCF are secreted by tumor cells, and produce matrix metalloproteinases (MMPs) [6]. Moreover, mast cells are a major source of histamine, which modulates tumor growth through H1 and H2 receptors [7]. H1 receptor antagonists significantly improved overall survival rates and suppressed tumor growth through the inhibition of hypoxia inducible factor-1 alpha (HIF-1α) expression in B16F10 melanoma-bearing mice [8]. Mast cells exert immunosuppression, releasing tumor necrosis factor alpha (TNF-α) and IL-10, which are essential in promoting the immune tolerance mediated by regulatory T (Treg) cells, and stimulate immune tolerance and tumor promotion [9,10].

Mast cells may promote inflammation, inhibition of tumor cell growth, and tumor cell apoptosis by releasing cytokines, such as IL-1, IL-4, IL-6, IL-8, monocyte chemotactic protein-3 and -4 (MCP-3 and MCP-4), transforming growth factor beta (TGF-β), and chymase. Finally, chondroitin sulphate may inhibit tumor cells diffusion and tryptase causes both tumor cell disruption and inflammation through activation of protease-activated receptors (PAR-1 and -2) [11].

## 3. Mast Cells and Tumor Angiogenesis

Mast cells release several pro-angiogenic factors, including fibroblast growth factor-2 (FGF-2), vascular endothelial growth factor (VEGF), IL-8, TNF-α, TGF-β, and nerve growth factor (NGF) [12,13,14,15,16,17,18,19,20,21]. Mast cells migrate in vivo and in vitro in response to VEGF and placental growth factor-1 (PlGF-1) [22,23,24]. In this context, VEGF may act both as an angiogenic factor and as an attractant factor for mast cells activating an autocrine loop of mast cell growth.

Human lung mast cells express VEGF-A, VEGF-B, VEGF-C and VEGF-D, and supernatants of activated lung mast cells induced angiogenic response in the chick embryo chorioallantoic membrane (CAM) assay that was inhibited by an anti-VEGF-A antibody [23]. Murine mast cells and their granules stimulate an angiogenic reaction in the CAM assay, partly inhibited by anti-FGF-2 and anti-VEGF antibodies [25]. Intraperitoneal injection of the compound 48/80 causes an angiogenic response in the rat mesentery window angiogenic assay and in mice [26,27]. Histamine and heparin stimulate proliferation of endothelial cells in vitro and are angiogenic in the CAM assay [28,29]. Mast cells store pre-formed active serine proteases in their secretory granules, including tryptase and chymase [30]. Tryptase stimulates the proliferation of endothelial cells, promotes vascular tube formation in vitro, and activates proteases, which in turn degrade the extracellular matrix with consequent release of VEGF or FGF-2 [31]. The expression of mast cell chymase and tryptase correlated with mast cell maturation and angiogenesis during tumor progression in chemically induced tumor growth in Bagg Albino (BALB)/c mouse [32]. Mast cells contain tissue inhibitors of metalloproteinases (TIMPs), [33,34] which intervene in regulation of extracellular matrix degradation, modulating the activation of angiogenic factors which is promoted by MMPs released by mast cells. Mast cell-deficient W/Wv mice exhibit a decreased rate of tumor angiogenesis [35]. Development of squamous cell carcinoma in a human papilloma virus (HPV) 16 infected transgenic mouse model of epithelia carcinogenesis provided support for the participation of mast cells in tumor growth and angiogenesis [36,37].

An increased number of mast cells have been demonstrated in angiogenesis associated with vascular tumors, as well as a number of haematological and solid tumors (Table 1), in which mast cell accumulation correlate with increased neovascularization, mast cell VEGF and FGF-2 expression, tumor aggressiveness and poor prognosis [38,39,40].

## 4. The Role of Mast Cells in Angiogenesis in Plasmocytoma 

Kumar et al. [69] demonstrated a high grade microvascular density in bone marrow samples of patients with solitary plasmocytomas and these patients were more likely to progress to MM and had a shorter progression-free survival compared with patients with low-grade angiogenesis. Naganuma et al. [70] demonstrated in two cases of solitary plasmocytoma of the skull that tumor cells express both VEGF and FGF-2. Nakayama et al. [71] demonstrated that plasmocytoma cells inoculated s.c. in nu/nuBALC/c mice gave rise to tumors that were significantly more rapidly and highly vascularized when inoculated together with mast cells, as compared to tumor cells alone. Double staining demonstrated co-localization of toluidine blue and angiopoietin-1 (Ang-1) providing evidence that mast cells within tumors are the source of Ang-1. Antibodies anti VEGF-A and anti-Tie-2/Fc individually and together significantly reduced tumor growth induced by plasmocytoma cells and mast cells, as compared to controls. Swelam and Tamini [72] demonstrated a significant higher microvascular density and VEGF expression in both endothelial and tumor cells in bone marrow biopsy specimens of MM as compared to solitary plasmocytoma. 

## 5. The Role of Mast Cells in Angiogenesis in Multiple Myeloma 

In 1994, we demonstrated for the first time that bone marrow microvascular density is significantly increased in MM compared to monoclonal gammopathies of undetermined significance (MGUS), and moreover in active vs. non-active MM [73]. Angiogenesis is induced by plasma cells via angiogenic factors released by the cells composing the tumor microenvironment and loss of angiostatic activity in MGUS [74].

Within the bone marrow microenvironment, bone marrow stromal cells, hematopoietic stem cells, fibroblasts, osteoblasts/osteoclasts, adipocytes, endothelial precursor cells, T lymphocytes, macrophages, and mast cells, increase the concentration of angiogenic factors and matrix degrading enzymes in the bone marrow microenvironment by direct secretion or following stimulation by myeloma cells or by endothelial cells through paracrine interaction [75].

**Figure 1:**
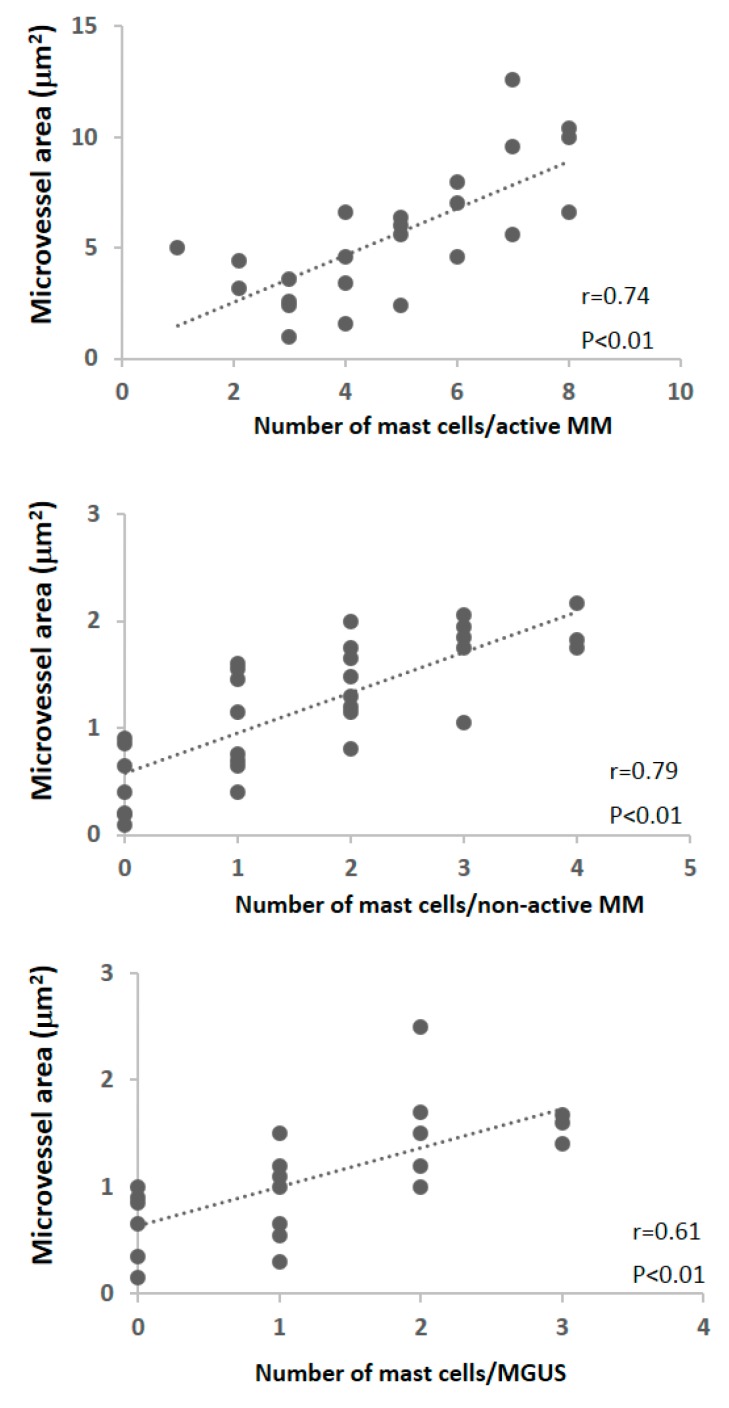
Mast cell counts in comparison with the microvessel area in the bone marrow of patients with active and non-active multiple myeloma (MM) and with monoclonal gammopathy of undetermined significance (MGUS). Significance of the regression analysis was calculated by the Pearson’s (r) test. (Modified from reference [46]).

**Figure 2:**
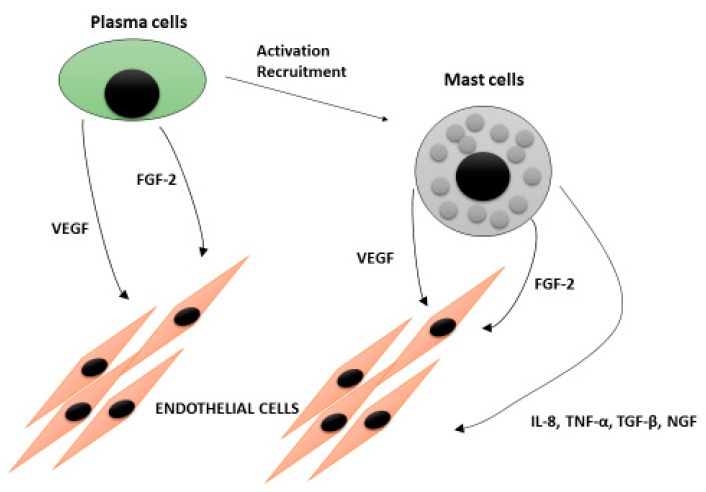
Interplay between plasma cells and mast cells in inducing angiogenic response in multiple myeloma.

**Figure 3:**
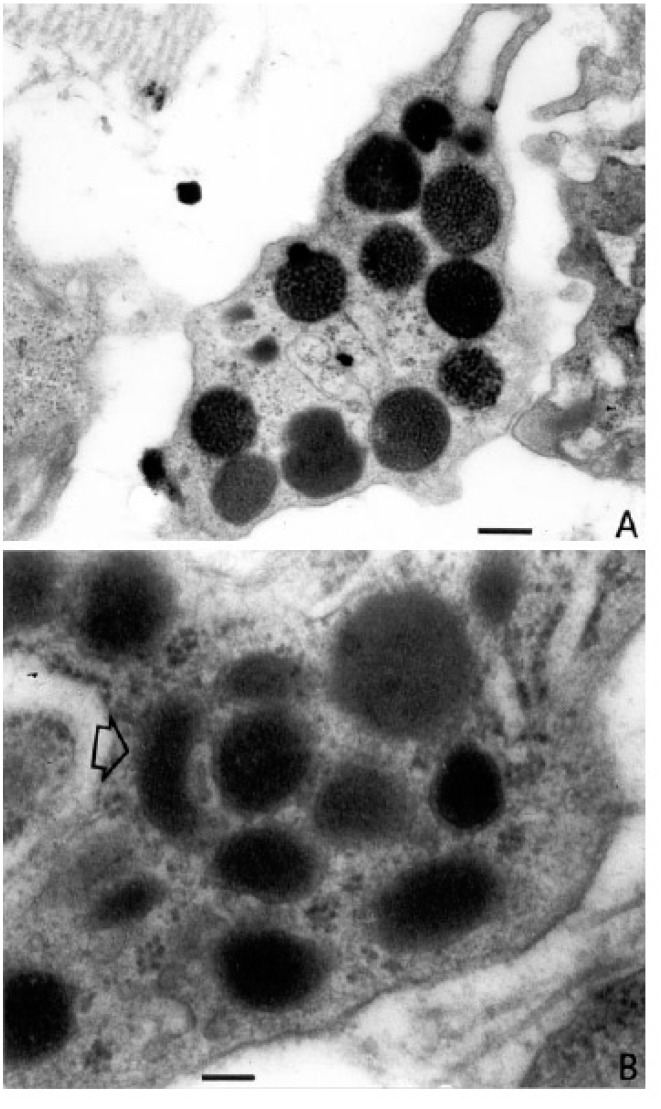
Ultrastructural findings of bone marrow biopsies from patients with active multiple myeloma. In (**A**), a mast cell with typical electron-dense round granules and in (**B**), at higher magnification, a cytoplasmic granule with a semilunar aspect (arrow), among other typical round granules, is recognizable. Bars, (**A**) 0.08 μm; (**B**) 0.02 μm (Reproduced from reference [46]).

We have shown that bone marrow angiogenesis and mast cell counts are highly correlated in patients with MM and in those with MGUS. In addition, both parameters increase simultaneously in active MM (Figure 1) [46]. These data suggest that an increasing number of mast cells may be recruited and activated by more malignant plasma cells in active multiple myeloma, and that angiogenesis in this disease phase may be mediated, at least in part, by angiogenic factors contained in their secretory granules (Figure 2) [76]. Interestingly, electron microscopic examination of bone marrow mast cells in MM patients shows ultrastructural features of slow and particulate secretion as it occurs in piecemeal degranulation (Figure 3) [46]. This ultrastructural appearance may reflect slow and progressive release of angiogenic factors by infiltrating mast cells, favoring chronic and progressive stimulation of mast cell degranulation.

**Figure 4:**
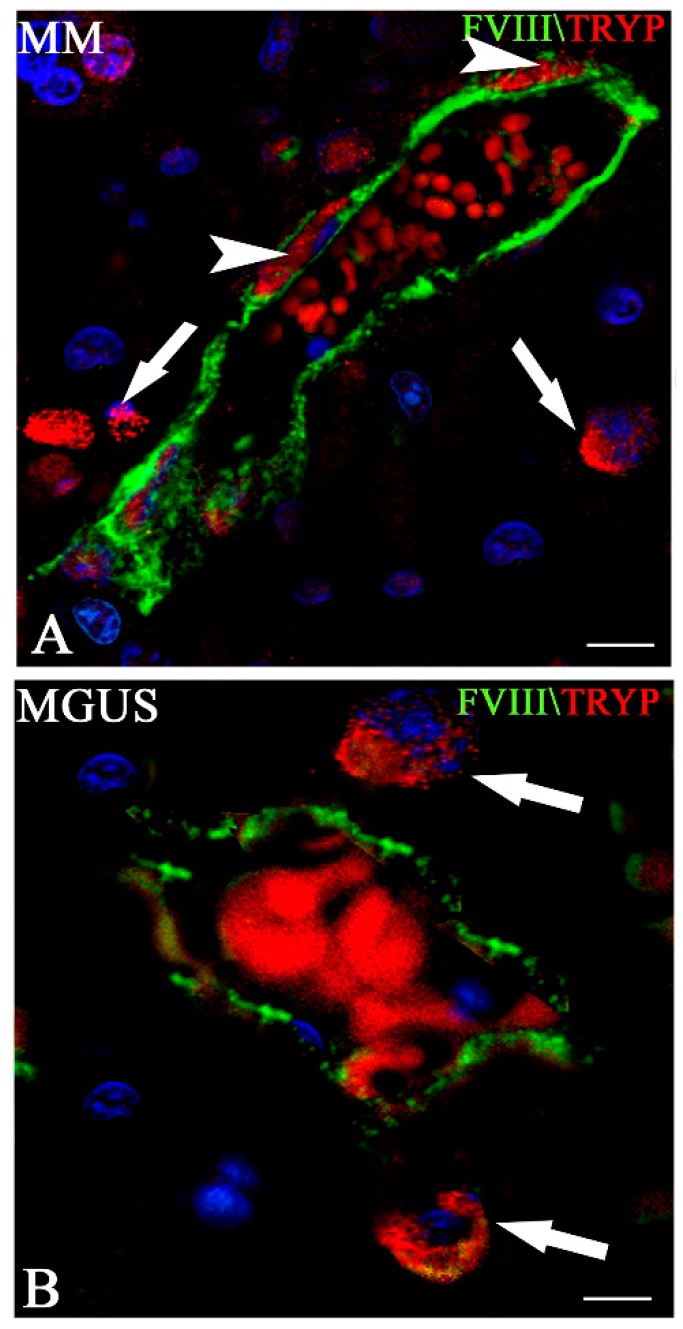
Double FVIII-RA (green) and tryptase (red) confocal laser microscopy from multiple myeloma (MM) (**A**) and monoclonal gammopathy of undetermined significance (MGUS) (**B**) bone marrow biopsy specimens. In (**A**), a MM vessel is lined by both endothelial cells positive for FVIII-RA and by mast cells positive for tryptase (arrowheads). Mast cells containing tryptase-positive granules (arrows) are also recognizable on the abluminal side of the vessel. In (**B**), a MGUS vessel is lined only by endothelial cells positive for FVIII-RA and is surrounded by tryptase-positive mast cells (arrows). Bars, (**A**) 15.8 μm; (**B**) 12.5 μm (Reproduced from reference [77]).

Besides stimulating angiogenesis in the bone marrow of MM patients, mast cells have the ability to contribute to vasculogenic mimicry [77]. We have demonstrated at ultrastructural level that vessels from MM bioptic specimens are lined by mast cells whose cytoplasm was filled by numerous and irregular shaped electron dense granules. Indeed, confocal microscopy approaches have demonstrated that in the bone marrow of patients with MM, typical tryptase-positive mast cells interact physically with the endothelial cells lining the vascular lumina, perhaps as a result of dysregulated vasculogenic development (Figure 4). 

This evidence highlights the importance of the stromal microenvironment during angiogenesis in the pathophysiology of MM and provides a novel perspective into the complex interplay between stromal and vascular components in the bone marrow microenvironment involved in the induction of hypervascularization. In contrast with this evidence, Mileshkin et al. [78] have found low or absent mast cell bone marrow infiltration in MM and an increase of mast cell number in patients in response to thalidomide, which has anti-angiogenic and immunomodulatory effects in myeloma.

Pappa et al. [79,80,81] showed that bone marrow mast cells density correlated with the clinical stage of MM and decreased after treatment; moreover, they quantified mast cells in bone marrow biopsies of MM patients and correlated with serum concentrations of VEGF, growth-regulated oncogene (GRO)-α, epithelial-derived neutrophil-activating peptide (ENA)-78. Mast cell and serum levels of GRO-α, ENA-78, and VEGF were significantly higher in MM patients compared to controls, and significant correlations were found between mast cell density with VEGF, GRO-α, ENA-78, and with a Ki-67 plasma cell proliferative index. Devetzoglou et al. [82] evaluated the mast cell density in bone marrow of untreated MM patients with markers of disease activity such as serum IL-6, B2M, and C-reactive protein (CRP), the grade of bone marrow infiltration, and the levels of produced paraprotein. The serum concentrations of CRP, B2M, and IL-6, and the mast cell density values were significantly higher in the MM patients’ group in comparison with those found in the control group. Significant differences were found between the grade of infiltration in bone marrow, and the paraprotein values in patients’ serum before and after chemotherapy. Furthermore, there was a significant correlation between the mast cell density values and the prognostic markers CRP, IL-6, bone marrow infiltration, and serum paraprotein. 

Vyzoukaki et al. [83,84] demonstrated that bone marrow mast cell density in MM patients is correlated with radiographic skeletal grades and evaluated serum levels of Ang-2 and MMP-9 and found them to be positively correlated with bone marrow mast cell density in patients with active MM.

## 6. Concluding Remarks and Therapeutic Perspectives

Bone marrow angiogenesis plays an important role in the pathogenesis and progression of hematological malignancies. Growth is halted and a dormancy state is induced in the avascular phase, whereas clonal expansion and epigenetic modifications of the microenvironment cause a switch to an angiogenic phenotype that generates the vascular phase, and involves changes in the local balance between pro- and antiangiogenic factors secreted by inflammatory cells and stromal cells.

The deregulated interactions between MM cells and other cells of the microenvironment are at the basis of the clinical manifestations of the disease, including osteolytic bone lesions, hypercalcemia, and suppressed haematopoietic functions. The vascular niche is comprised of vasculature forming a conduit which enables MM cells both to leave the osteoblastic niche and to enter the vascular system via transendothelial migration, hence to return to the bone marrow via homing mechanisms. 

In the treatment of MM, thalidomide has been part of the standard treatment for and is thought to inhibit VEGF-associated angiogenesis, while Bevacizumab, a monoclonal antibody directed against VEGF-A, inhibits VEGF, as well as the proteasome inhibitor bortezomib. Accordingly, the efficacy and safety of these agents alone and in combination in MM patients have been tested [85,86,87].

The new targeted anti-cancer therapies may exert effects on mast cells (Table 2). Many of these drugs have mast cells c-kit as the main target, and inhibition of the SCF/Kit axis in vivo inhibits the migration of bone marrow-derived cultured mast cells to tumors. Chemoprevention with an anti-inflammatory approach has the potential to inhibit neovascularization before the onset of the angiogenic switch, resulting in a significant delay in tumor growth. Moreover, the development of novel therapies to alter mast cell function in the tumor microenvironment could inhibit tissue remodeling and tumor growth and activate the immune system. 

In this context, mast cells might act as a new target for the adjuvant treatment of haematological tumors through the selective inhibition of angiogenesis, tissue remodeling and tumor-promoting molecules, permitting the secretion of cytotoxic cytokines and preventing mast cell-mediated immune suppression. 

## Figures and Tables

**Table 1:** Tumors in which a relationship between angiogenesis and mast cell number has been established.

Tumor	References
Haemangioma, haemangioblastoma	[41]
Lymphomas	[42,43,44,45]
Multiple myeloma	[46]
Bagg AlbinoMyelodysplastic syndrome	[47]
B-cell chronic lymphocytic leukemia	[38,48]
Breast cancer	[49,50,51,52,53]
Gastric cancer	[54,55,56,57]
Colorectal cancer	[58,59]
Pancreatic cancer	[60,61]
Uterine cervix cancer	[62,63,64]
Melanoma	[39,65,66]
Pulmonary adenocarcinoma	[67]
Thyroid cancer	[68]

**Table 2:** Anti-tumor drugs that target regulatory mast cell molecules.

Drug	Main Target in Mast Cell
Imatinib mesylate (Gleevec, ST1571)	c-kit
Sorafenib	c-kit
Sunitinib	c-kit
Pazopanib (GW786034)	c-kit
Axitinib	c-kit
Dasatinib	c-kit
Enzastaurin	PKC-β
Alemtuzumab (Campath)	CD52
CpG activator (Promune)	TLR9
MDX 060	CD30 (ligand for CD301) *
Tanespimycin (17AAG)	Heat shock protein 90 β

* Which can regulate chemokine secretion by mast cells.
